# Role of Anti-B-Cell Maturation Antigen (BCMA) in the Management of Multiple Myeloma

**DOI:** 10.3390/cancers14143507

**Published:** 2022-07-19

**Authors:** Ikhwan Rinaldi, Abdul Muthalib, Brenda Cristie Edina, Lowilius Wiyono, Kevin Winston

**Affiliations:** 1 Division of Hematology and Medical Oncology, Department of Internal Medicine, Ciptomangunkusumo General Hospital—Faculty of Medicine, Universitas Indonesia, Jakarta 10430, Indonesia; amuthalib@hotmail.com; 2 Faculty of Medicine, Universitas Indonesia, Jakarta 10430, Indonesia; brenda.cristie@ui.ac.id (B.C.E.); lowilius.wiyono@alumni.ui.ac.id (L.W.); kevinwinston4@gmail.com (K.W.); 3 Hospital Medicine, Bhakti Medicare Hospital, Cicurug 43359, Indonesia

**Keywords:** multiple myeloma, cancer, BCMA

## Abstract

**Simple Summary:**

Multiple myeloma is the most prevalent hematological cancer, and further treatments for this disease are required. Despite progress in the development of treatment regimens, multiple myeloma is still an incurable disease because of its poor response to therapy and high rate of resistance to treatment. However, anti-BCMA (B-cell maturation antigen) has shown promise in the treatment of multiple myeloma, and it may have the potential to be a new first-line treatment for patients. Thus, in this review, we objectively discussed the treatment potential of anti-BCMA, its mechanisms, and its future clinical implications for multiple myeloma patients.

**Abstract:**

Over the past few decades, treatment options have become more advanced for multiple myeloma (MM), one of the most prevalent hematological cancers; however, multiple myeloma remains an incurable disease due to its poor response to therapy and high rates of resistance, which cause relapsed/refractory or multiple myeloma. Researchers have described anti-BCMA (B-cell maturation antigen) as a promising treatment regimen that targets the BCMA biomarker in the affected plasma cells. BCMA is a protein that is specifically expressed in plasma-cell neoplasms by using several mechanisms, such as CAR T cells (Chimeric Antigen Receptor T cells), antibody-drug conjugates, and bispecific T-cell engagers, thus allowing for a rapid response in the treatment of resistant or relapsed/refractory multiple myeloma patients. Anti-BCMA treatment is novel and specific in its mechanisms of action, with noninferior complete responses, higher overall survival rates, and fewer reported adverse events compared to other currently available treatment of MM. In this review, we compared anti-BCMA mechanisms with those of previously available therapies, such as those using immunomodulators and proteasome inhibitors, and discussed the advantages of using anti-BCMA as a potential first-line treatment for multiple myeloma patients.

## 1. Introduction

Multiple myeloma, a proliferative clonal plasma-cell neoplasm, is one of the most prevalent hematological cancers in the world [1,2,3]. Due to its abnormal proliferation and excessive immunoglobulin production, multiple myeloma may cause progressive end-organ damage, which leads to a poor prognosis, especially for high-risk populations [3].

The treatment modalities of multiple myeloma have advanced over the past four decades, especially because the use of proteasome and histone deacetylase inhibitors, immunomodulatory imide drugs, and monoclonal antibodies has been approved [4,5], which have succeeded in doubling the 5-year survival rate of patients [1]. However, the 5-year survival rate of multiple myeloma patients in high-risk populations is around 50% or lower [4].

Despite advancements in therapy, multiple myeloma remains an incurable disease due to its high rates of resistance to treatment, which can result in relapsed/refractory multiple myeloma [3,4,5]. The complete, partial, and overall responses of patients to current therapy (e.g., proteasome inhibitors) still vary; furthermore, a fraction of patients have short, or no, durations of response to therapy [5]. Some classes of treatment, such as monoclonal-antibody-targeted therapy, are still not specific enough to target active cancer cells and thus also target other hematopoietic lineage and immune effector cells, causing increased morbidity in long-term clinical use [6]. An unsatisfactory treatment response leads to the inevitable development of relapsed/refractory multiple myeloma (RRMM), resulting in shorter durations of disease remission and resistance to standard salvage therapies [4,5,6].

Patients younger than 65 years can be eligible for a stem cell transplant, which decreases early mortality rates in the three years after diagnosis, produces a longer duration of complete response, and decreases relapse rates compared with chemotherapies [7]. However, the success rates of allogeneic stem cell transplants are low, resulting in high mortality and relapse rates post-transplantation, with a 3-year overall and progression-free survival rate of 76.7% [8].

Novel approaches to multiple myeloma therapy are still needed to provide better treatment responses and decrease morbidity and resistance to therapy in long-term use. A potential target molecular biomarker for MM therapy is the B-cell maturation antigen (BCMA), which is expressed in normal mature B cells and not in other tissues, overly expressed in multiple myeloma, and plays a role in disease severity, progression, and relapse [4,5,6]. Previous findings about the use of anti-BCMA in multiple myeloma have shown promising results for clinical effectivity regarding the use of drug-antibody conjugates, CAR T cells, bispecific T-cell engagers, and/or bispecific or trispecific antibodies [6]. BCMA-targeted therapies increased responses in patients that had failed therapies with anti-CD38 antibodies, proteasome inhibitors, and immunomodulatory agents [6].

Therefore, we conducted a thorough literature review to provide better comparisons between the potential of BCMA-targeted and standard therapy, especially in their mechanisms of action, clinical efficacy, and safety.

## 2. Multiple Myeloma

### 2.1. Burden of Disease

Multiple myeloma is characterized by an abnormal increase in monoclonal antibodies. It is the second most prevalent hematological cancer in the world, with an incidence of 1.8 cases per 100,000 persons and a mortality rate of 1.1 per 100,000 persons due to its specific end-organ damage [1,2,3]. In southeast Asia, which comprises generally developing countries, its incidence and mortality rates are lower, at 0.96/100,000 and 0.82/100,000, respectively [2]. Predictions indicate that the frequency of cases regarding the disease is set to increase by 80% per year, with the number of cases doubling in the next 20 years because of the aging population and the prevalence of MM in elderly patients [3,9]. Multiple myeloma is more debilitating in high-risk populations, including frail elderly patients, who have a median age of 70 years at diagnosis, and patients with high-risk cytogenetics and RRMM [3,4,9].

### 2.2. Pathogenesis

The etiology of multiple myeloma is possibly genetic abnormalities in oncogenes (CMYC, NRAS, and KRAS) associated with other external factors (obesity; drinking alcohol; insecticide, organic solvent, and radiation exposure); however, its exact causes are still unknown. Differences in molecular and genomic profiles exist in the tumor clones of MM. Hypodiploidy and 14q32 translocations are two of the most commonly observed cytogenetic abnormalities in MM. The affected chromosome contains λ light-chain regions and κ light-chain genes [3].

Cytogenetic abnormality during an immune response to abnormal antigenic challenge results in abnormal asymptomatic premalignant clonal plasma-cell growth and monoclonal antibody production, termed monoclonal gammopathy of undetermined significance (MGUS) [3,10]. Next, MGUS develops into smoldering multiple myeloma due to the additional ‘second hit’ cytogenetic abnormality when asymptomatic and then to overt myeloma when symptomatic [3,10]. Excessive circulating monoclonal antibodies, including heavy and light chains, result in the clinical manifestation of overt multiple myeloma, which leads to hyperviscosity, platelet and neurologic dysfunction, bleeding, and renal tubular damage and failure [3,10].

### 2.3. Therapy

Six classes of pharmacological therapy are currently available for the treatment of MM, namely, proteasome (e.g., carfilzomib, bortezomib, and ixazomib) and histone deacetylase inhibitors (panobinostat), monoclonal antibodies (daratumumab and elotuzumab), immunomodulatory drugs (lenalidomide and pomalidomide), DNA-alkylating agents, and glucocorticosteroids [11]. Mono or combination therapy using proteasome inhibitors, immunomodulatory drugs, and monoclonal antibodies is recommended depending on the purpose of chemotherapy and patient characteristics. EHA-ESMO (2021) recommended that fit patients eligible for autologous stem cell transplant and aggressive therapy undergo induction therapy using VRd (bortezomib + lenalidomide + dexamethasone) or DaraVTD (daratumumab + bortezomib + melphalan + prednisone), which is to be continued with melphalan for ASCR adjuvant and lenalidomide for maintenance [12]. EHA-ESMO (2021) recommended DaraRd (daratumumab + lenalidomide + dexamethasone), DaraVMP (daratumumab + bortezomib + thalidomide + dexamethasone), or VRd as induction therapy for patients ineligible for ASCT or those <70 years old [13].

## 3. Role of BCMA in MM Pathogenesis

BCMA is a transmembrane glycoprotein expressed on normal B cells and is abundantly found in malignant plasma cells. It is a 184-amino-acid chain with a conserved pattern of six cysteines in the extracellular N terminus, and it is encoded by a 2.92 kb TNFRSF17 gene in the short arm of chromosome 16 (16p13.13) [14]. BCMA is a tumor necrosis factor (TNFR superfamily, which also includes the B-cell-activating factor receptor (BAFF-R or BLyS-R)), and it is also a transmembrane activator, calcium modulator, and cyclophilin ligand interactor (TACI) [4,14]. Members of the TNFR superfamily work together in the proliferation, maturation, survival, and differentiation of B cells from plasma cells [4,11]. BCMA is activated by its agonist ligands, APRIL and BAFF. APRIL binds to both BCMA and TACI, whereas BAFF binds to BAFF-R and BCMA [4,14]. The overexpression of BCMA and its ligands in MM activates both the classical and alternative nuclear factor kappa B (NFκB)-signaling pathways, which result in increased gene expression for growth, survival, adhesion, angiogenesis, metastasis, osteoclast activation, and immunosuppression [4,14]. In contrast, TACI and BAFF predominantly trigger the classical and alternative NFκB pathways, respectively [11].

The superiority of BCMA compared with other TNFR superfamily members (BAFF and TACI) lies in its specificity in expression: it is only expressed on plasmablasts and plasma cells [4,14]. Memory B and plasmacytoid dendritic cells, and other organs, such as the testis, trachea, and gastrointestinal duct, also display minimal expression. Naïve B and hematopoietic stem cells, as well as other nonhematologic tissues, do not express BCMA [4,14]. Another main advantage of BCMA is that the glycoprotein is found on 80–100% of MM cell lines, more so in malignant plasma cells compared with normal plasma cells, and it is distinct from TACI and BAFF-R, which are almost undetectable or detected in lower concentrations [4,14]. BCMA is an ideal biomarker specifically for MM diagnosis because of these characteristics.

The results of previous studies have also shown the potential of BCMA in monitoring disease progression, treatment response, and the prognostication of MM. Regarding disease progression, a linear increase in BCMA levels was observed during its progression from MGUS and SMM to overt MM [15]. Soluble BCMA (sBCMA), which is BCMA circulating in plasma directly shed from the BCMA membrane, is a marker correlated with an increased risk of progression to MM if found in higher levels in MGUS or SMM patients [16]. Correlations also exist between higher levels of BCMA and poorer treatment outcomes, especially in patients with sBCMA levels above 25–325 ng/mL [17,18]. sBCMA levels declined in patients with a positive response to therapy [12], which also positively correlated with the clinical manifestations and tumor-mass sizes recorded in preclinical studies [12,14]. BCMA also had a shorter half-life of 24 to 26 h compared with the levels of currently available M-proteins, which could assist in more rapidly reflecting the disease status [14].

## 4. Anti-BCMA

B-cell maturation antigen (BCMA) is one of the promising targets for immunotherapy in the management of relapsed/refractory multiple myeloma. Researchers have hailed BCMA (tumor necrosis factor receptor superfamily member 17 (encoded by the TNFRSF17 gene)) as one of the most prominent factors in the survival of B cells, alongside the commonly used B-cell-activating factor receptor (BAFF-R) and the responsible proliferation-inducing ligand (APRIL). Early trials of the role of BCMA were conducted in vivo, where the overexpression of BCMA in MM cell lines activated the protein kinase pathway, which led to a promotion in MM-cell growth. An overexpression of BCMA levels in MM cell lines also positively correlated with its interactions with factors involved in cell proliferation. In a study of the KMS12 MM cell line, BCMA co-immunoprecipitated with interferon regulatory factor 4, which mediates cell survival. APRIL and BAFF also promoted cell growth and survival when binding with BCMA and the transmembrane and cyclophilin ligand activators and calcium modulator (TACI), which further activated the NFκB pathways and increased the expression of antiapoptotic proteins (Mcl-1, Bcl-xL, and Bcl-2) that protect the MM cells from serum-deprivation-induced cell death and dexamethasone. Both APRIL and BAFF are critical factors in the survival of plasma cells in multiple myeloma because their levels considerably increase in both ligands and receptors in MM patients compared with normal subjects. Thus, creating novel therapies that target BCMA and the influential factors it interacts with are useful developments in treating multiple myeloma [6].

**Figure 1:**
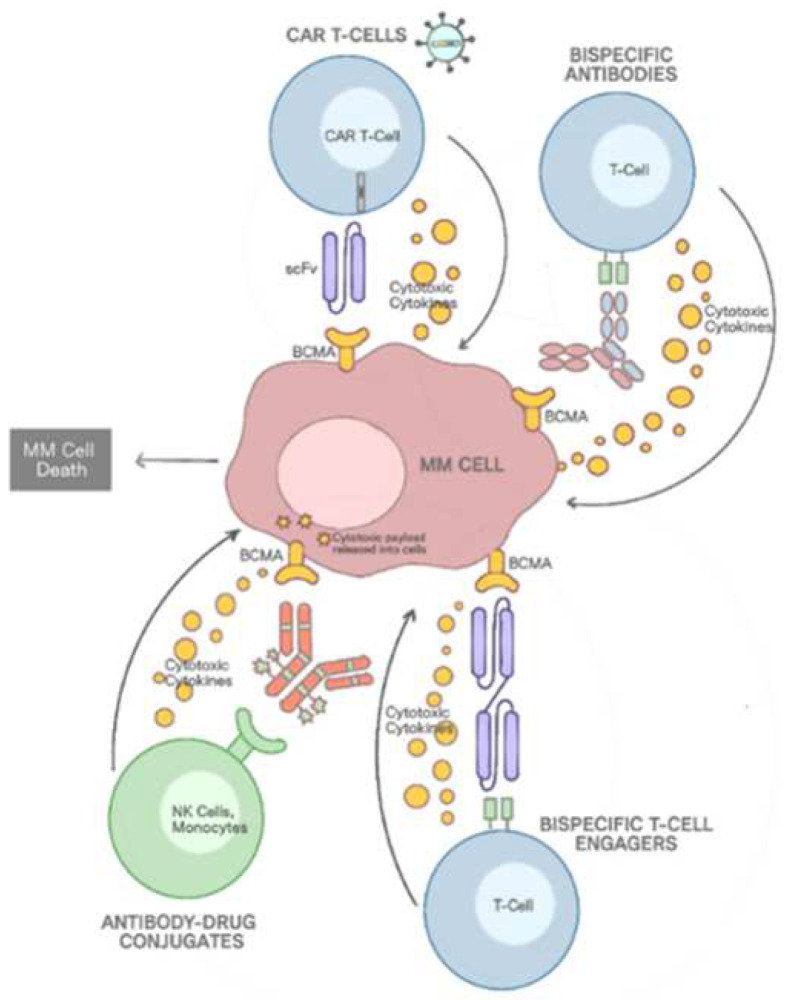
Anti-BCMA-therapy mechanism for multiple myeloma.

The growing awareness of the BAFF/APRIL/BCMA axis has popularized BCMA as one of the major targets in the management of multiple myeloma. Three main immunotherapy treatments now exist, namely, antibody-conjugated drugs (ACD), the bispecific T-cell engager (BiTE) or the anti-BCMA antibody, and chimeric antigen receptor (CAR) T-cell therapies, as shown in Figure 1. The three therapies have their own mechanisms for hindering the upregulation of antiapoptosis signals in MM cells [19].

## 5. BCMA-ADC (Antibody-Drug Conjugate)

BCMA-ADC is mainly used to enhance antibody-dependent cellular toxicity (ADCC). Binding BCMA-ADC to the BCMA receptor disrupts BAFF and APRIL signaling, which consequently decreases the number of NFκB pathways and the expression of anti-apoptotic proteins. Antibody-drug conjugates also bind with FcyIIa receptors in natural killer (NK) cells, inducing ADCC, which directly causes MM cell apoptosis. Belantamab mafodotin (GSK2857916), which works by being coupled to the microtubule inhibitor monomethylauristatin F (MMAF) using the protease-resistant maleimidocaproyl linker, is the most prominent type of ADC used. MMAF is a byproduct of the monoclonal antibody backbone of the drug after it is proteolytically degraded upon exposure to lysosomes released from MM cells undergoing ADCC-induced apoptosis. MMAF is then able to bind with tubulin in MM cells, which is involved in the assembly of microtubules, halting the G2/M checkpoint of the cell cycle and preventing the proliferation of MM cells [19,20].

## 6. Bispecific T-Cell Engager (BiTEs)

Clinicians have used T-cell-based antibody therapy in recent years, including T-cell-engaging bispecific antibodies (T-BsAbs) or BiTEs. BiTEs use a dual-targeting antibody mechanism to enable each arm of the antibody to bind to another receptor. The mechanism of action involves one arm of the antibody binding to the CD3 coreceptor complex on T cells, while the other arm binds to the tumor cells through the tumor-associated antigen, namely, BCMA. The binding of the T cell produces the cytolytic-initiating protein (perforin) to induce the cytotoxicity process, which produces transmembrane pores on the tumor cells to initiate apoptosis [19,20].

## 7. Chimeric Antigen Receptor (CAR) T Cells

CAR T cells are also therapeutic agents that use the T cell-based antibody mechanism. In recent years, the use of CAR T cells has increased in cancer immunotherapies, including in the treatment of multiple myeloma. The manufacturing of CAR T cells involves the use of adoptive cell transfer (ACT) to convert patient-derived cytotoxic T cells into specific killers of cancer cells. Patient-derived cytotoxic T cells become CAR T cells when they undergo recombinant DNA mutation, so that they express a chimeric receptor against the antigens of targeted cancer cells. The single-chain variable fragment (ScFv) on the CAR T cells’ chimeric receptor is specific to BCMA, which enables its binding only to BCMA-expressing plasma cells, leading to the activation of the CAR T cells and the release of cytotoxic cytokines that target the MM cells to which they bind [20].

CAR T cells are commonly used in the management of multiple myeloma; however, the therapy itself, due to the limited quantities of trial results, is now being used as a fourth-line treatment for relapsed/refractory multiple myeloma. In particular, monoclonal antibodies or ADC are only provided if the patient is considered unresponsive to chemotherapy [13]. Researchers have hypothesized that CAR T cells are effective in the treatment of other hematological cancers, for which they also showed considerably successful results, in particular with B-cell malignancies. However, attempts were also made to incorporate the therapy for the treatment of other malignancies, including solid tumors [21].

## 8. Comparison of the Mechanism of Action to Currently Available Therapies

**Figure 2:**
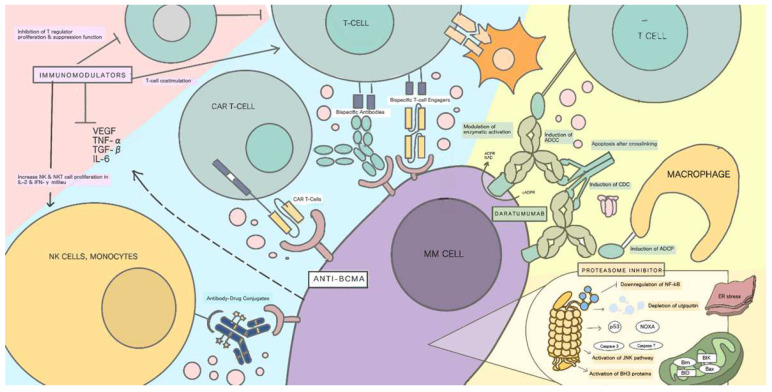
Known therapies for multiple myeloma and their mechanisms of action.

We highlight the potential of anti-BCMA therapy through a comparison with the mechanisms of other therapies for multiple myeloma, as described in Table 1 and pictured in Figure 2. Each of the available therapies target various receptors and antigens and display different processes.

### Mechanism of Action

Regarding the mechanisms of action in other classes, proteasome inhibitors induce malignant plasma cell death through the inhibition of NFκB-signaling pathways, especially by preventing the degradation of the kappa B (IκBα) inhibitor in proteasomes, which leads to the failure of p50/p65 NFkB transcription-factor activation and its translocation to the nucleus [11,22]. Proteasome inhibitors have other known mechanisms, such as the activation of the JNK pathway and caspases, and interference in the degradation of proapoptotic proteins, which leads to the apoptosis of malignant plasma cells [11,22].

Immunomodulators act by reversing the immunoparesis caused by MM, enhancing the adaptive immune response targeting cancer cells [25]. Immunomodulators augment T-cell costimulation, inhibit plasma cell-derived cytokines, inhibit T-regulator proliferation and suppressor functions, and increase NK- and NKT-cell proliferation in the IL2- and IFN-gamma milieu [29]. We also found that immunomodulators displayed direct cytotoxic activity toward myeloma cells through the production of increased proapoptotic and decreased antiapoptotic factors, the inhibition of NFκB and PI3K/Akt pathways, and the downregulation of proteins involved in producing interferon regulatory factors [26].

Monoclonal antibodies act through their identification of various antigens, usually specific proteins expressed by malignant cells, and induce cell death through direct cytotoxic effects and cellular cytotoxicity mediated by antibodies (ADCC) and complements (CDC) [28,29]. Daratumumab is the most commonly used monoclonal antibody in the treatment of myeloma, and it targets a transmembrane glycoprotein called CD38, which is expressed by plasma, lymphoid, and myeloid cells and other nonhematopoietic tissues. Daratumumab induces cytotoxicity in MM cells through ADCC-, ADCP-, CDC-, and FcR-mediated cross-linking and its modulation of enzymatic activation [28,29].

Compared with the proteasome inhibitors, immunomodulators, and other monoclonal antibodies mentioned above, the novelty of anti-BCMA lies in its specificity toward target antigens in target cells and in its more direct response toward plasma cells. This is because anti-BCMA has a mechanism that targets BCMA on the surface of malignant plasma cells while also binding to other immune effectors to provide a direct response and induce cellular death. It takes a different approach compared with other multiple myeloma therapies, such as proteasome inhibitors, which work on modulating the endoplasmic reticulum of the MM cells; immunomodulators (IMIDs), which modulate T cells; NK cells, which identify MM cells; or other drugs, such as daratumumab, which causes MM cells to be easily recognized by immune cells. Furthermore, anti-BCMA targets an earlier phase of MM pathogenesis before the activation of the NFκB-signaling pathway. Depending on the type of anti-BCMA, the reaction of the targeted pathway varies from direct cytotoxicity to the stimulation of a cancer-specific immune response, providing more alternatives to battle the mechanisms of drug resistance.

## 9. Anti-BCMA Potential for Multiple Myeloma

The use of anti-BCMA in the management of multiple myeloma has been incorporated into the current guidelines, which entail the use of this treatment, combined with the nonresponsive use of triple therapy beforehand, for relapsed/refractory multiple myeloma [13]. Trials concerning the use of anti-BCMA have been ongoing for each type of drug that targets the BCMA. However, because most studies have only progressed to phase 2 of clinical trials, they used single- or double-arm trials, with different doses as their control. Patients with relapsed/refractory multiple myeloma and who had used several regimens beforehand were the most common sample in these studies.

In the clinical trial utilizing monoclonal antibodies or ADC, belantamab mafodotin showed a considerably high overall response rate (60%) in phase 1. In phase 2 (DREAMM-2), different doses of belantamab mafodotin were compared (2.5 vs. 3.4 mg/kg) and produced an overall response rate of 31% and 34%, respectively, which showed the limited effectivity of the drug. The drug also increased the rates of adverse events in the subjects, who reported keratopathy (27%), thrombocytopenia (20%), anemia (20–47%), and corneal events (blurry vision and dry eyes) [33]. Belantamab mafodotin was then combined with another agent (pembrolizumab) in the DREAMM-4 clinical trial, which showed increasing overall response rates, with 67% and 14% reported for the 2.5 and 3.4 mg/kg doses, respectively, and with a similar trend for adverse events [30]. Alternative agents of ADC were also tested in a trial, namely, MEDI2228 and HDP-101; however, no known reports have been produced [14].

Trials using bispecific antibodies on T cells, with an overall response rate of 70% and a median response time of one month, reported the use of AMG420 with a recommended dose of 400 ug/kg as an effective bispecific antibody for BCMA and CD3. However, serious adverse events, such as polyneuropathy and cytokine-release syndrome, hospitalized 18 patients (out of 42), and the researchers also reported patients suffering from edema [31]. Another trial, using BiTes, was performed with CC-93269, with an overall response rate of 83.3% and a dose of >6 mg, and resulted in several adverse events, such as CRS (89%), neutropenia (55%), anemia (26%), and thrombocytopenia (89%). Regarding CRS management, most patients were diagnosed with grade-1 CRS, which was considered manageable [32]. Humanized BCMA and CD3 BiTes were developed, namely, PF-3135, and although no serious adverse event was reported, the clinical benefit rate was 41%, with 35% of patients displaying stable disease [34,35]. Additionally, BCMAxCD3 BiTes was also developed, called REGN5458; however, the results of the study reported that one out of three patients had stable disease [36].

On the development of CAR T cells as an anti-BCMA, several therapies showed considerable efficacy in the early phases of clinical trials. While some had similarities, most of the CAR T cells differed because of the costimulatory domains and species used to generate the anti-BCMA domain on the T cell. The recorded overall response rate ranged from 48% to 86%, which showed the considerable effectivity of CAR T cells in the treatment of patients; furthermore, while several adverse effects were reported (mostly neuro- and nephrotoxicity), no serious complications were reported as related to the therapy itself. The CAR T cells currently being evaluated in clinical trials can be further classified into various CAR T products [37,38,39,40].

The classical anti-BCMA CAR T cells are commonly represented by bb2121, which contains an anti-BCMA single-chain fragment, a CD8 hinge, and transmembrane domains, which consist of a single transduction region composed of a CD3 chain and the costimulatory molecule 4-1BB [37]. The trial with the therapy showed stringent complete (36%), complete (9%), considerable partial (27%), and partial response rates (12%). Products in this classification include KITE-585 CT053, with 100% overall and complete response rates in 2/13 patients [38], and CT103A, which also had 100% overall and 66% complete response rates [39]. bb2121 was further developed and bb21217 was created, to which 6/7 patients had positive clinical responses after they used a therapy dose of 150 × 10^6^ cells [40]. In these classical anti-BCMA CAR-T therapies, most patients experienced hematologic toxicity, such as neutropenia (85%), leukopenia (58%), anemia (45%), and thrombocytopenia (45%). CRS was also diagnosed in most patients (76%), and most were diagnosed (70%) as grade 1 or 2. The bb2121 trial results showed that 3% of samples had a reversible grade-4 neurologic toxic effect; however, other trials reported no such adverse effect [37,38,39,40].

CAR T cells can also be classified as bispecific anti-BCMA CAR-T products, which means that they can modify T cells, with one CAR molecule containing two distinct binding domains that commonly target both BCMA and APRIL receptors. The products under development are APRIL-based CAR, such as ACAR, TriPRIL CARm, and LCAR-B38M CAR T cells (bi-epitope anti-BCMA CAR-T cells). Bispecific CAR T cells showed an improvement in the complete response rate (68% complete response with LCAR-B38M) [41]. The dual-target BM38 was also developed and showed an 87.5% overall response rate, with 12.5% showing a complete response [42]. Other variations of CAR T cells include compounds, such as anti-BCMA CAR T, the CAR T-containing PI3K inhibitor and cetyrin, and the optimized-spacer-, safety-switch-, or autologous-mRNA-generated anti-BCMA CAR T [21]. Similar to the classical BCMA, most patients had hematologic toxicity and CRS, with most only diagnosed with grade 1 or 2 CRS and no hospitalization needed.

The currently developed humanized CAR T cells, such as anti-BCMA CD19 and MCARH171 (human-derived CAR T cells), have also undergone phase-1 clinical trials. The overall response rate was 64%, with CRS observed in 6/11 patients and 1 diagnosed with encephalopathy. A dose–response relationship was also evaluated, with promising clinical efficacy for CAR T cells at dose levels ≥ 450 × 10^6^ [43,44].

The use of anti-BCMA has shown considerable efficacy in the management of multiple myeloma, while the monotherapy of CAR T cells has shown low complete response rates; however, the therapy has the potential to be used in combination with another immunotherapy available for multiple myeloma. Although no direct comparison was made between the use of anti-BCMA and the usual regimen of multiple myeloma, the regimen using rituximab, lenalidomide, and bendamustine showed a complete response rate of 64%, which was higher compared with anti-BCMA [32]. However, the results of a case series also reflected the failure of CAR-T-cell therapy, which might be explained by the increase in anti-CAR-T-cell antibodies on the second infusion of the treatment [34]. The treatment can be used as maintenance therapy due to its minimal adverse effects. However, further trials might be needed to determine the use of anti-BCMA for transplant-eligible and -ineligible patients.

## 10. Conclusions

The expression of BCMA in MM cells highlights the potential for improvements in diagnosis, disease prognosis, and treatment–response control. Anti-BCMA could provide an alternative therapy for MM and alleviate the current challenges posed by inadequate treatment response and disease treatment intolerance and resistance. We showed anti-BCMA to be novel and specific in its mechanism of action and found it to produce a noninferior complete response and an improvement in overall survival rates, as well as a decrease in the numbers of reported adverse events. Based on our review, we determined that anti-BCMA has the potential to be used as a combination therapy in conjunction with currently available therapy, for both initial MM and RRMM.

## Figures and Tables

**Table 1:** Summary of MM therapies.

Class	Drugs	Target of Action	Mechanism of Action	Reported Toxicities	Indication
Proteasome inhibitor	Bortezomib Carfilzomib Ixazomib	Proteasomes of malignant plasma cells	Inhibition of IκBα (classical pathway) degradation in proteasome [11,22]; Activation of JNK pathway and caspases [11,22]; Inhibition of pro-apoptotic protein degradation [11,22]	Peripheral neuropathy, nausea, vomiting, diarrhea, cytopenia, infection, fatigue, headache, peripheral edema, and back pain [23,24]	Initial induction therapy (bortezomib) [13,23,24]; Recurrent/relapsed therapy (carfilzomib, ixazomib) [23,24].
Immunomodulators	Thalidomide Lenalidomide	B and T lymphocytes Malignant plasma cells	Augmentation of T-cell costimulation [25]; Inhibition of plasma-cell-derived cytokines [25]; Inhibition of T-regulator proliferation and suppressor function [25]; Increasing NK- and NKT-cell proliferation in IL2- and IFN-gamma milieu [25]; Direct cytotoxicity to malignant cells through apoptosis pathways [26]	Cytopenia, infection, fatigue, and peripheral neuropathy [27]; Deep-vein thrombosis (in combination with dexamethasone [27]	Induction and maintenance therapy [25,26]; Recurrent/relapsed [25,26]
Monoclonal antibodies	Daratumumab Elotuzumab	Surface antigens of malignant plasma cells, CD38 (daratumumab), and CS1/SLAMF7 (elotuzumab)	Antibody-dependent cellular cytotoxicity [28,29]; Complement-dependent cellular cytotoxicity [28,29]		Induction therapy [13]; Recurrent/relapsed MM [24,27]
Anti-BCMA	ADC (belantamab mafodotin)	BCMA	Coupling to MMFA [20,22]; Direct cytotoxicity [20,22]	Thrombocytopenia, anemia, and corneal events [30]	Recurrent/relapsed MM [13]
	BiTEs	BCMA	Binding to T cells and induction of apoptosis through perforin [20,22]; Direct cytotoxicity [20,22]	No serious adverse events reported yet [31]	
	CAR T cells	BCMA	Conversion of patient-derived cytotoxic T cells into specific killers of cancer cells using recombinant DNA mutation process [25,26]	Neurotoxicity, nephrotoxicity [32]	
